# Correlation analysis of psychiatric and behavioral symptoms with cognitive impairment and prognosis in patients with general paresis

**DOI:** 10.3389/fpsyt.2026.1769770

**Published:** 2026-04-16

**Authors:** Taosha Meng, Yaoqin He, Lina Qin, Qiping Liang, Zhangmin Luo, Xiaofeng Zhang, Chuyang Li, Qiwei Li, Fengkun Zhou, Xianfeng Li

**Affiliations:** 1 Department of Neurology, The Fifth Affiliated Hospital of Guangxi Medical University, Nanning, Guangxi, China; 2 Department of Internal Medicine, The Fourth People’s Hospital of Nanning, Nanning, Guangxi, China; 3 Department of Internal Medicine, Guangxi Medical University, Nanning City, Guagnxi, China

**Keywords:** cognitive impairment, GP patients, influencing factors, prognosis, psychiatric and behavioral symptoms

## Abstract

**Objective:**

To investigate the characteristics of neuropsychiatric symptoms (NPS) in patients with general paresis (GP) and analyze their correlation with cognitive impairment and prognosis.

**Methods:**

Clinical data from 100 GP patients admitted between January 2022 and June 2024 were retrospectively analyzed. Neuropsychiatric Inventory (NPI) assessed neuropsychiatric symptoms, while Mini-Mental State Examination (MMSE) evaluated cognitive function. All patients received standardized anti-syphilis treatment and completed 12-month follow-up. Based on cerebrospinal fluid test results at the final follow-up, patients were categorized into good and poor prognosis groups. Spearman correlation analysis explored the relationship between NPS and cognitive impairment, while multivariate logistic regression identified independent risk factors for poor prognosis.

**Results:**

All 100 patients exhibited varying degrees of NPS. The mean NPI total score was (42.38 ± 29.96) points, with depression (6.20 ± 3.83) and apathy (5.75 ± 3.78) scoring highest, while aberrant motor behavior (0.62 ± 1.08) scored lowest. All patients exhibited cognitive impairment, with a mean MMSE score of (13.78 ± 2.92). Impaired recall ability (0.94 ± 0.69) was the most significant deficit. Spearman correlation analysis revealed a significant negative correlation between NPI total scores and MMSE total scores (r = -0.583, P < 0.001). Additionally, all NPI subscales (e.g., delusions r = -0.573, appetite and eating changes r = -0.556) showed significant negative correlations with MMSE total scores (all P < 0.001). At the 12-month follow-up, 38 patients (38.00%) had a good prognosis, while 62 patients (62.00%) had a poor prognosis. Multivariate logistic regression analysis indicated that lower educational attainment (OR = 0.191, 95% CI: 0.052–0.709, P = 0.013), elevated baseline cerebrospinal fluid white blood cell count (OR = 3.194, 95% CI: 1.628–6.268, P<0.001), higher NPI total scores (OR = 1.073, 95% CI: 1.029–1.119, P<0.001), and lower MMSE scores (OR = 0.493, 95% CI: 0.338–0.720, P<0.001) were independent risk factors for poor prognosis in GP patients.

**Conclusion:**

The severity of psychiatric and behavioral symptoms showed a significant negative correlation with the degree of cognitive impairment. GP patients with low educational attainment, high baseline cerebrospinal fluid white blood cell counts, severe psychiatric and behavioral symptoms, and poor cognitive function had a higher risk of poor prognosis. Clinicians should develop targeted interventions addressing these high-risk factors to improve patient outcomes.

## Introduction

General paresis (GP) represents a severe manifestation of tertiary neurosyphilis. It arises from *Treponema pallidum* invading the central nervous system, with extensive cortical involvement as its core pathological feature. Clinically, it manifests as progressive cognitive decline, personality changes, and psychiatric behavioral abnormalities, posing a grave threat to patients’ neurological and social functioning (1, 2). Although the widespread use of penicillin-based drugs has significantly reduced the incidence of GP, global syphilis infection rates have shown a resurgence in recent years. Compounded by the insidious early symptoms of GP and its high clinical overlap with diseases such as Alzheimer’s disease and schizophrenia, rates of misdiagnosis and missed diagnosis remain high. This leads to some patients missing the optimal window for intervention (3, 4), and the disease burden remains significant.

Clinical manifestations in GP patients are complex and variable, with cognitive impairment being a common symptom. Patients often exhibit memory decline, slowed reactions, and diminished abilities in temporal orientation, personal orientation, and calculation, severely impacting daily life and work (1). Additionally, neuropsychiatric symptoms (NPS) are another frequent clinical feature, further complicating the disease presentation. Patients may exhibit depressive symptoms like low mood and loss of interest, or psychotic symptoms such as delusions of persecution and hallucinations. Some also display behavioral abnormalities including impulsive aggression and nocturnal behavioral disturbances (3, 5). These symptoms not only directly diminish patients’ quality of life but also increase psychological and financial burdens on caregivers. Furthermore, they may impair treatment adherence (e.g., medication refusal, avoidance of follow-up visits), thereby compromising the efficacy of anti-syphilis therapy and creating a vicious cycle of “symptom exacerbation → treatment disruption → disease progression” (6, 7).

Currently, research on GP has primarily focused on diagnostic criteria, pathological mechanisms, and antithymidylate synthase therapy regimens (1), while systematic studies examining the association between neuropsychiatric symptoms and cognitive impairment remain scarce. In particular, quantitative analyses of the impact of neuropsychiatric symptoms on disease prognosis are notably lacking. Clarifying the intrinsic link between NPS and cognitive impairment not only provides new clinical indicators for assessing GP disease severity but also offers evidence-based support for developing comprehensive treatment strategies—combining anti-infective therapy, symptom intervention, and long-term management—by identifying high-risk factors for poor prognosis.

Therefore, this retrospective analysis of 100 GP patients aims to systematically describe NPS occurrence patterns, investigate its correlation with cognitive impairment, and identify independent risk factors for poor prognosis. This study seeks to fill existing research gaps and provide evidence for precision clinical management of GP.

## Materials and methods

### Study population

This retrospective study included patients with GP hospitalized in the Department of Neurology of The Fifth Affiliated Hospital of Guangxi Medical University and Department of internal medicine, The Fourth People’s Hospital of Nanning, from January 2022 to June 2024.

Inclusion Criteria: 1. Meets the diagnostic criteria for paralytic dementia outlined in the Expert Consensus on Diagnosis and Treatment of Neurosyphilis: presence of progressive cognitive impairment or psychiatric abnormalities, positive treponemal-specific antibodies (TPPA) in serum and cerebrospinal fluid (CSF), positive non-specific syphilis serology (RPR/TRUST) in CSF, and elevated CSF white blood cell count (>5×10^6^/L); 2. Age between 18 and 75 years; 3. Complete clinical records and follow-up data; 4. Ability to complete required assessment tools such as the Neuropsychiatric Inventory (NPI) and Mini-Mental State Examination (MMSE).

Exclusion Criteria: 1. Concurrent other types of dementia, such as Alzheimer’s disease, vascular dementia, or Lewy body dementia; 2. Presence of severe organic brain disorders such as traumatic brain injury, cerebrovascular accident, or brain tumor; 3. History of diagnosed primary psychiatric disorders including schizophrenia, bipolar disorder, or paranoid psychosis; 4. Concurrent systemic diseases such as severe hepatic or renal insufficiency, malignancy, or severe cardiopulmonary dysfunction; 5. Inability to complete questionnaire assessments due to impaired consciousness or severe communication barriers, or voluntary withdrawal from the study during follow-up for personal reasons.

Based on the above inclusion and exclusion criteria, 100 patients were ultimately selected as study subjects, all of whom completed the 12-month follow-up. This study was reviewed and approved by our hospital’s ethics committee and complies with the Declaration of Helsinki.

**Figure 1:**
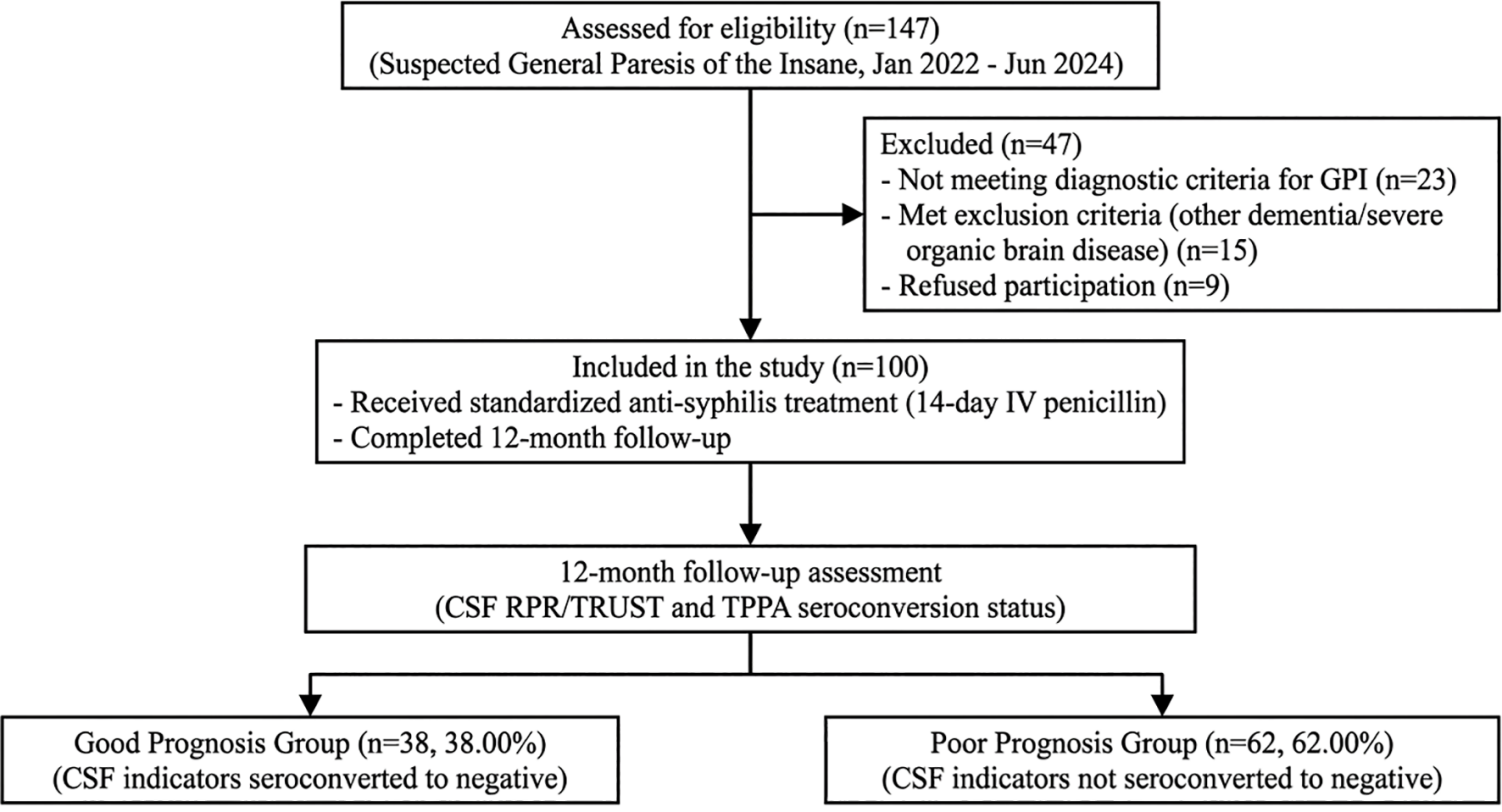
STROBE flowchart of participant selection and follow-up. STROBE flowchart of participant selection, inclusion, and follow-up. A total of 147 patients with suspected general paresis (GP) were initially screened between January 2022 and June 2024. After applying inclusion and exclusion criteria, 47 patients were excluded (23 did not meet diagnostic criteria for GP, 15 met exclusion criteria including other types of dementia or severe organic brain disorders, and 9 declined participation). The remaining 100 patients were enrolled in the study, received standardized anti-syphilis treatment, and completed the 12-month follow-up period. At final follow-up, based on cerebrospinal fluid (CSF) test results (RPR/TRUST and TPPA conversion), patients were categorized into good prognosis (n = 38) and poor prognosis (n = 62) groups.

In accordance with the STROBE (Strengthening the Reporting of Observational Studies in Epidemiology) guidelines, **Figure 1** presents a flowchart detailing the selection process of study participants. The flowchart illustrates the number of individuals assessed for eligibility, excluded patients with reasons, the final cohort included in the analysis, and their distribution into prognostic groups at 12-month follow-up.

### Data collection and extraction procedures

Data were retrospectively collected from the electronic medical record system by two independent investigators (QPL and ZML) between July and September 2024. A standardized data extraction form was used to ensure consistency. The following demographic and baseline clinical information was extracted for each patient from the first available records after hospital admission: Demographic Information: Patient age (in years), gender (male/female), educational attainment, and marital status. Educational attainment was classified into three levels based on the highest completed grade: elementary school or below (0–6 years of education), junior high to high school (7–12 years of education), and college or above (>12 years of education). Marital status was recorded and categorized as married, unmarried, or divorced/widowed. Baseline Laboratory Test Results: The following results were extracted from the first specimen (blood and cerebrospinal fluid) collected after hospital admission: Qualitative results for serum *Treponema pallidum* particle agglutination assay (TPPA) (positive/negative). Qualitative results for cerebrospinal fluid (CSF) *Treponema pallidum* particle agglutination assay (TPPA) (positive/negative). CSF white blood cell count, measured as cells × 10^6^/L.

To ensure data accuracy, all extracted data were independently cross-checked by a third investigator (XFZ). Any discrepancies were resolved through discussion and re-examination of the original medical records.

### Evaluation indicators

#### Assessment of neuropsychiatric symptoms

The Neuropsychiatric Inventory (NPI) is used to evaluate patients’ neuropsychiatric symptoms (8). The scale comprises 12 symptom dimensions: delusions, hallucinations, agitation, depression, anxiety, euphoria, apathy, disinhibition, irritability, aberrant motor behavior, nighttime behavioral disturbances, and appetite and eating changes. Each dimension is scored based on “frequency of occurrence (0 = absent, 1 = <1 time per week, 2 = 1–3 times per week, 3 = 4–6 times per week, 4 = daily)” and “severity (0 = absent, 1 = mild, 2 = moderate, 3 = severe).” The final score for each symptom dimension = Frequency score × Severity score. The NPI total score is the sum of scores across all 12 dimensions, ranging from 0 to 144 points. Higher scores indicate greater overall severity of neuropsychiatric symptoms.

#### Cognitive impairment assessment

The Mini-Mental State Examination (MMSE, Chinese version) is used to evaluate patients’ cognitive function (9). The scale comprises six assessment dimensions: time orientation (5 points), spatial orientation (5 points), memory (3 points), attention and calculation (5 points), recall (3 points), and language (9 points). The MMSE total score ranges from 0 to 30 points, with higher scores indicating better cognitive function.

### Prognosis follow-up and assessment

All patients received standardized anti-syphilis treatment (following the Expert Consensus on Diagnosis and Treatment of Neurosyphilis, involving intravenous penicillin therapy for 14 days) and underwent 12-month follow-up. Follow-up methods included outpatient visits (every 3 months) and telephone follow-ups (monthly), with the final follow-up occurring 12 months after treatment completion.

At the final follow-up visit, cerebrospinal fluid (CSF) samples were collected again to test CSF RPR/TRUST and TPPA indices. Prognosis assessment criteria were as follows: Poor prognosis was defined as failure of CSF RPR/TRUST to convert, failure of TPPA to convert, or failure of both to convert. Based on these criteria, the 100 patients were divided into a good prognosis group (n=38) and a poor prognosis group (n=62).

### Statistical analysis

Data statistical analysis was performed using SPSS 27.0 statistical software (SPSS, Inc., Chicago, IL, USA) and GraphPad Prism 9.50 software (GraphPad Software Inc., San Diego, CA, USA). The Shapiro-Wilk test was used to assess data normality. Normally distributed quantitative data were expressed as mean ± standard deviation and analyzed using the independent samples t-test. Non-normally distributed quantitative data were expressed as interquartile ranges and compared using the Mann-Whitney U test. Categorical data were presented as counts and percentages [n (%)] and analyzed using the chi-square test. Spearman correlation analysis was used to explore the relationship between neuropsychiatric symptoms (NPI total score and dimension scores) and cognitive function (MMSE total score). A *post hoc* statistical power analysis was conducted using G*Power software (version 3.1.9.7). With an alpha level of 0.05, a sample size of 100, and an odds ratio of 1.073 for the NPI total score (the smallest effect size among significant predictors), the achieved power for the logistic regression model was 0.94, indicating adequate sample size to detect the observed effects. Multivariate logistic regression analysis was employed to identify independent risk factors for poor prognosis in patients with GP. Variables with P < 0.05 in univariate analysis were included as independent variables, with the prognostic outcome (good = 0, poor = 1) as the dependent variable. To assess multicollinearity among independent variables, variance inflation factor (VIF) was calculated for each predictor. All VIF values were below 2.5 (NPI: 1.89, MMSE: 1.92, CSF WBC: 1.34, education: 1.21), indicating no significant multicollinearity concerns. The Hosmer-Lemeshow goodness-of-fit test was also performed to evaluate the overall model fit, yielding a chi-square value of 6.84 with 8 degrees of freedom (P = 0.554), indicating adequate model calibration. A P value < 0.05 was considered statistically significant.

## Results

### Baseline characteristics

This study included 100 patients with GP. All patients met the diagnostic criteria for GP outlined in the Expert Consensus on Diagnosis and Treatment of Neurosyphilis and completed a 12-month follow-up. Baseline patient characteristics were distributed as follows: age range 37~70 years, mean age (54.60 ± 6.14) years. Male patients numbered 85 (85.00%), while female patients numbered 15 (15.00%). Educational attainment distribution was: primary school or below 26 (26.00%), junior high to high school 48 (48.00%), and 26 cases (26.00%) with college education or higher, with the highest proportion being patients with secondary education. Regarding marital status, 75 cases (75.00%) were married, 12 cases (12.00%) were unmarried, and 13 cases (13.00%) were divorced or widowed, with married patients constituting the main population. All patients tested positive for *Treponema pallidum*-specific antibodies (TPPA) in serum, and cerebrospinal fluid (CSF) non-specific syphilis serological tests (RPR/TRUST) were positive. The mean CSF white blood cell count was (6.84 ± 1.40) × 10^6^/L.

### Characteristics of psychiatric behavioral symptoms in patients

The Neuropsychiatric Inventory (NPI) was used to assess psychiatric behavioral symptoms (NPS) in 100 patients. Results indicated that all patients exhibited varying degrees of NPS, with specific characteristics as follows (**Table 1**): NPI total scores ranged from 8 to 103 points, with a mean score of (42.38 ± 29.96) points. Symptom dimension scores, ranked from highest to lowest, were: Depression (6.20 ± 3.83) and apathy (5.75 ± 3.78) had the highest scores, with 91.00% (91/100) of patients exhibiting symptoms in both dimensions; followed by anxiety (5.04 ± 3.70) (86 cases, 86.00%), irritability (4.77 ± 3.98) (73 cases, 73.00%), nighttime behavioral disturbances (4.53 ± 3.42) (87 cases, 87.00%), delusions (4.05 ± 3.45) points (73 cases, 73.00%), hallucinations (3.56 ± 3.01) points (70 cases, 70.00%), agitation (3.13 ± 2.91) points (68 cases, 68.00%); disinhibition (2.12 ± 2.86) points (49 cases, 49.00%), appetite and eating changes (1.52 ± 2.06) points (48 cases, 48.00%), euphoria (1.09 ± 1.78 points, 39 cases, 39.00%) scored relatively lower, while aberrant motor behavior (0.62 ± 1.08 points) scored the lowest, present in only 31 cases (31.00%). This indicates that depression and apathy are the most prominent psychiatric behavioral issues in patients with GP, whereas abnormal motor behavior is relatively uncommon in this patient population.

**Table 1:** Characteristics of psychiatric and behavioral symptoms in patients with GP.

Psychiatric symptom dimension	Number of symptomatic cases (n, %)	Average score (points)
Delusions	73(73.00%)	4.05 ± 3.45
Hallucinations	70(70.00%)	3.56 ± 3.01
Agitation	68(68.00%)	3.13 ± 2.91
Depression	91(91.00%)	6.20 ± 3.83
Anxiety	86(86.00%)	5.04 ± 3.70
Euphoria	39(39.00%)	1.09 ± 1.78
Apathy	91(91.00%)	5.75 ± 3.78
Disinhibition	49(49.00%)	2.12 ± 2.86
Irritability	73(73.00%)	4.77 ± 3.98
Aberrant Motor Behavior	31(31.00%)	0.62 ± 1.08
Nighttime Behavioral Disturbances	87(87.00%)	4.53 ± 3.42
Appetite and Eating Changes	48(48.00%)	1.52 ± 2.06
NPI Total Score		42.38 ± 29.96

### Characteristics of cognitive impairment in patients

Cognitive function was assessed in all patients using the Chinese version of the Mini-Mental State Examination (MMSE). The MMSE is a 30-point scale that evaluates six cognitive domains: time orientation (5 points), spatial orientation (5 points), immediate memory (3 points), attention and calculation (5 points), delayed recall (3 points), and language (9 points). Higher scores indicate better cognitive function, with a total score of ≤24 generally suggesting cognitive impairment.

Results indicated that all patients exhibited varying degrees of cognitive impairment, with an average MMSE total score of (13.78 ± 2.92) points, which falls within the moderately severe range of cognitive decline. A detailed analysis of the subdomain scores (**Table 2**) revealed a pattern of widespread impairment. Scores across all cognitive domains fell below corresponding normal ranges, with recall ability scoring the lowest at an average of (0.94 ± 0.69) points (normal range: 3 points); followed by attention and calculation (2.16 ± 1.08 points; normal range: 5 points), time orientation (2.23 ± 0.94 points; normal range: 5 points), and spatial orientation (2.43 ± 1.12 points) (normal 5 points), and memory (1.88 ± 0.90 points) (normal 3 points). Language ability showed relatively higher but still significantly impaired scores, averaging (4.14 ± 1.56 points) (normal 9 points). The most profound deficits were observed in delayed recall and attention/calculation, while language, although impaired, was comparatively less affected. Overall, this reflects widespread and multidimensional cognitive impairment in GP patients, with a pattern suggestive of predominant fronto-temporal-subcortical involvement.

**Table 2:** Spearman correlation analysis of psychiatric symptoms and cognitive impairment.

Psychiatric symptom dimension	Correlation coefficient (r) with MMSE total score	Score
Delusions	-0.573	<0.001
Hallucinations	-0.412	<0.001
Agitation	-0.483	<0.001
Depression	-0.519	<0.001
Anxiety	-0.529	<0.001
Euphoria	-0.503	<0.001
Apathy	-0.468	<0.001
Disinhibition	-0.510	<0.001
Irritability	-0.525	<0.001
Aberrant Motor Behavior	-0.526	<0.001
Nighttime Behavioral Disturbances	-0.534	<0.001
Appetite and Eating Changes	-0.556	<0.001
NPI Total Score	-0.583	<0.001

### Correlation between psychiatric behavioral symptoms and cognitive impairment

Spearman correlation analysis was employed to examine the association between NPI total scores and individual symptom dimensions with MMSE total scores. Results (**Table 2**) demonstrated a significant negative correlation between NPI total scores and MMSE total scores (r = -0.583, P < 0.001), indicating that more severe psychiatric behavioral symptoms correlate with greater cognitive impairment. All behavioral and psychological symptom dimensions showed significant negative correlations with MMSE total scores (all P<0.001). Given that 13 simultaneous correlations were performed, a Bonferroni correction was applied to adjust for multiple comparisons, setting the significance threshold at P < 0.0038 (0.05/13). All reported correlations remained statistically significant after this adjustment, confirming the robustness of the findings. Ranked by absolute correlation coefficient magnitude from highest to lowest: delusions (r = -0.573), appetite and eating changes (r = -0.556), nighttime behavioral disturbances (r = -0.534), anxiety (r = -0.529), aberrant motor behavior (r = -0.526), irritability (r = -0.525), depression (r = -0.519), disinhibition (r = -0.510), euphoria (r = -0.503), agitation (r = -0.483), apathy (r = -0.468), and hallucinations (r = -0.412). Among these, delusions showed the strongest association with cognitive impairment (approaching the strength of the NPI total score), while hallucinations exhibited relatively weaker associations. However, all dimensions reached the level of “significant negative correlation,” indicating a “generalized association” between psychotic and behavioral symptoms and cognitive impairment in GP patients, rather than being confined to specific symptom domains.

**Figure 2:**
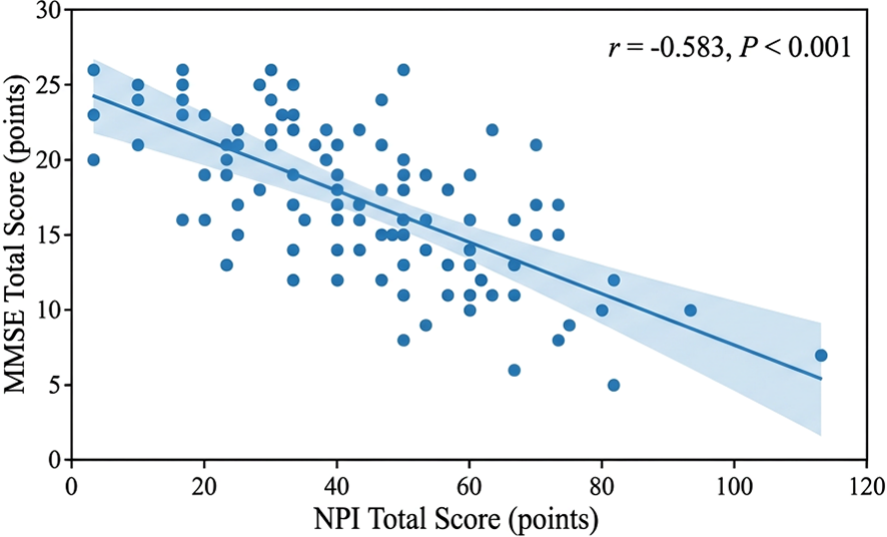
Scatterplot of the correlation between NPI total scores and MMSE total scores. Scatterplot showing the correlation between Neuropsychiatric Inventory (NPI) total scores and Mini-Mental State Examination (MMSE) total scores in 100 patients with general paresis (GP). Each point represents an individual patient. The blue line represents the linear regression fit with 95% confidence bands (shaded area). Spearman’s correlation analysis revealed a significant negative correlation (r = -0.583, P < 0.001), indicating that more severe neuropsychiatric symptoms are associated with poorer cognitive function.

To visually illustrate the relationship between neuropsychiatric symptom severity and cognitive function, **Figure 2** presents a scatterplot of NPI total scores against MMSE total scores. The figure includes individual data points, a fitted linear regression line with 95% confidence bands, and the Spearman correlation coefficient.

### Patient prognosis

All patients received standardized anti-syphilis treatment (following the Expert Consensus on Diagnosis and Treatment of Neurosyphilis, involving intravenous penicillin therapy for 14 days) and completed 12-month follow-up. Based on cerebrospinal fluid (CSF) test results at the final follow-up (considered good prognosis if both CSF RPR/TRUST and TPPA turned negative; poor prognosis if either remained positive), patients were categorized into a good prognosis group (38 cases, 38.00%) and poor prognosis group (62 cases, 62.00%). Comparison of baseline clinical characteristics between groups (**Table 3**) showed no statistically significant differences in age, gender, or marital status (all *P* > 0.05). However, statistically significant differences were observed in educational attainment, baseline CSF white blood cell count, NPI total score, and MMSE total score (all *P* < 0.05). Specifically, the good prognosis group exhibited higher educational attainment (36.84% with college education or higher), lower CSF WBC counts, lower NPI total scores, and higher MMSE total scores.

**Table 3:** Comparison of baseline clinical characteristics among patients in different prognosis groups.

Variable	Good prognosis group (n=38)	Poor prognosis group (n=62)	t/χ2/Z	*P*
Age	54.32 ± 5.93	54.77 ± 6.31	0.361	0.719
Gender (n, %)				
Male	33 (86.84%)	52 (83.87%)	0.163	0.686
Female	5 (13.16%)	10 (16.13%)		
Educational Attainment (n, %)				
Elementary school or below	5 (13.16%)	21 (32.26%)	6.710	0.035
Junior high to high school	19 (50.00%)	29 (46.77%)		
College or above	14 (36.84%)	12 (20.97%)		
Marital Status (n, %)				
Married	29 (76.32%)	46 (74.19%)	0.057	0.812
Unmarried/Divorced/Widowed	9 (23.68%)	16 (25.81%)		
Cerebrospinal Fluid White Blood Cell Count	6.17 ± 1.20	7.25 ± 1.36	4.021	<0.001
NPI Total Score	10.00 (8.00, 32.00)	56.00 (30.00, 81.25)	-6.218	<0.001
MMSE Total Score	16.50 (15.00, 18.00)	12.00 (11.00, 14.00)	-6.271	<0.001

The Shapiro-Wilk test was used to assess the normality of the data distribution. Normally distributed quantitative data were expressed as mean ± standard deviation, with intergroup comparisons performed using the independent samples t-test. Non-normally distributed quantitative data were presented as quartiles, with intergroup comparisons conducted using the Mann-Whitney U test. Count data were expressed as counts and percentages, analyzed using the chi-square test. P < 0.05 was considered statistically significant.

### Logistic regression analysis of factors affecting poor prognosis in GP patients

To evaluate factors influencing poor prognosis in GP patients, we conducted univariate logistic regression analysis using age, gender, education level, marital status, cerebrospinal fluid (CSF) white blood cell count, NPI total score, and MMSE total score—baseline clinical characteristics from patients in different prognosis groups (**Table 4**)—as independent variables, with prognosis outcome as the dependent variable (good = 0, poor = 1). The specific coding methods for each variable are detailed in **Table 4**. Analysis results indicated that educational attainment, CSF WBC count, NPI total score, and MMSE score were significantly associated with poor prognosis in GP patients (all P < 0.05) (**Table 5**). Subsequently, variables with P < 0.05 were included in a multivariate logistic regression analysis. Results (**Table 5**) indicated that lower educational attainment (OR = 0.191, 95% CI: 0.052–0.709, *P* = 0.013), high baseline cerebrospinal fluid white blood cell count (OR = 3.194, 95% CI: 1.628–6.268, *P* < 0.001), higher NPI total scores (OR = 1.073, 95% CI: 1.029–1.119, *P* < 0.001), and lower MMSE scores (OR = 0.493, 95% CI: 0.338–0.720, *P* < 0.001) were independent risk factors for poor prognosis in patients with GP. Sensitivity analyses were conducted by sequentially adjusting for age and gender in separate models; the odds ratios for the four main predictors remained stable (changes <10%), indicating that these factors were not confounders.

**Table 4:** Assignment table for research variables.

Variables	Variable type	Assignment specifications
Prognosis Outcomes	Categorical Variable	Good = 0, Poor = 1
Age	Continuous Variable	Actual Measurement Value
Gender	Categorical Variable	Male = 1, Female = 0
Educational Attainment	Categorical Variable	Dummy-coded with “Elementary school and below” as reference: Dummy1 (Junior high to high school = 1, others = 0); Dummy2 (College and above = 1, others = 0)
Marital Status	Categorical Variable	Married = 1, Unmarried/Divorced/Widowed = 0
Cerebrospinal Fluid White Blood Cell Count	Continuous Variable	Actual Measurement Value
NPI Total Score	Continuous Variable	Actual Measurement Value
MMSE Total Score	Continuous Variable	Actual Measurement Value

**Table 5:** Logistic regression analysis of factors associated with poor prognosis in GP patients.

Variable	Single-factor analysis			Multivariate analysis			VIF	Effect size (Cohen’s d)
	*P*-value	OR-value	95%CI	*P-*value	OR-value	95%CI		
Age	0.716	1.012	0.948~1.082	–	–	–		
Gender	0.687	0.788	0.247~2.510	–	–	–		
Educational Attainment	0.012	0.463	0.253~0.845	0.013	0.191	0.052~0.709	1.21	0.52
Marital Status	0.812	0.892	0.349~2.283	–	–	–		
Cerebrospinal Fluid White Blood Cell Count	<0.001	1.917	1.332~2.761	<0.001	3.194	1.628~6.268	1.34	0.78
NPI Total Score	<0.001	1.067	1.040~1.096	<0.001	1.073	1.029~1.119	1.89	0.91
MMSE Total Score	<0.001	0.512	0.394~0.666	<0.001	0.493	0.338~0.720	1.92	0.85

To provide complete transparency regarding the univariate analysis, **Supplementary Table 1** presents the full univariate logistic regression results for all candidate variables assessed as potential predictors of poor prognosis in GP patients. This table includes unadjusted odds ratios, 95% confidence intervals, and P-values for age, gender, educational attainment, marital status, baseline cerebrospinal fluid white blood cell count, NPI total score, and MMSE total score.

## Discussion

Central nervous system damage caused by *Treponema pallidum* infection exhibits insidious and progressive characteristics. As a severe subtype of late neurosyphilis, GP poses clinical hazards not only through direct destruction of brain parenchyma by *Treponema pallidum* but also through the long-term impact of multidimensional functional disorders—specifically “cognitive–psychological–behavioral” multidimensional functional disorders on patients’ quality of life and long-term treatment outcomes (10, 11). Despite the standardization of current syphilis treatment protocols, clinical practice reveals that some patients fail to achieve serological conversion in cerebrospinal fluid after standardized therapy, with persistent neuropsychiatric symptoms and cognitive impairment, suggesting that disease prognosis may be regulated by multiple factors (12, 13). Through retrospective analysis of clinical data from 100 GP patients, this study systematically reveals the distribution characteristics of neuropsychiatric symptoms (NPS), confirms their negative correlation with cognitive impairment, and identifies independent risk factors for poor prognosis. These findings provide evidence-based support for developing a comprehensive clinical strategy combining “anti-infective therapy + symptom intervention + high-risk management.” In recent years, increasing attention has been directed toward the glymphatic system—a brain-wide perivascular network responsible for clearing metabolic waste, inflammatory mediators, and neurotoxic proteins—as a potential mechanism linking neuroinflammation, cognitive decline, and psychiatric symptoms. This emerging perspective offers a novel framework for interpreting the multidimensional clinical features observed in GP.

First, this study found that all GP patients exhibited varying degrees of neuropsychiatric abnormalities, with an average NPI total score as high as (42.38±29.96) points, indicating the high prevalence and severity of neuropsychiatric symptoms in this disease. Pathologically, GP results from *Treponema pallidum* invasion of the central nervous system. Its core pathological changes involve chronic inflammation and neuronal degeneration in the cerebral cortex, particularly affecting the frontal lobe, temporal lobe, and limbic system—the frontal lobe regulates emotions and social behavior, while the temporal lobe and limbic system participate in emotional processing and motivational regulation (14). Emerging evidence suggests that chronic neuroinflammation may also impair glymphatic function, a perivascular network critical for clearing metabolic waste and inflammatory by-products from the brain. In the context of GP, persistent inflammation could compromise glymphatic clearance, exacerbating neuronal damage and contributing to the widespread cortical dysfunction observed clinically. Among these, depression and apathy were the most prominent symptoms, both occurring at a rate of 91.00% and ranking highest in scores across all dimensions. This finding aligns with previous research on the pathological mechanisms by which neurosyphilis causes frontal-limbic dysfunction—*Treponema pallidum* directly damages neurons and induces immune inflammatory responses, leading to neuronal degeneration and synaptic dysfunction in these brain regions. This not only causes cognitive impairment but also disrupts emotional regulation and motivational maintenance, manifesting as symptoms like depression and apathy (1, 15, 16). Furthermore, the prevalence of anxiety, nocturnal behavioral disturbances, irritability, and delusions exceeded 70%, indicating a broad spectrum of psychiatric symptoms in GP patients encompassing affect, perception, and behavioral regulation (3, 17). In contrast, euphoria, disinhibition, and abnormal motor behaviors occurred less frequently, potentially reflecting a clinical phenotype dominated by “inhibitory” rather than “excitable” psychiatric disorders in this cohort. Notably, despite standardized anti-syphilis treatment, 62% of patients failed to achieve serological conversion in cerebrospinal fluid at the 12-month follow-up. This suggests that current treatment strategies have limited efficacy for some patients, highlighting the urgent need to identify high-risk populations and intensify interventions (18).

Second, all patients exhibited significant cognitive impairment, with a mean MMSE score of (13.78±2.92), far below the normal reference range (typically ≥24 points), indicating that GP is essentially a syndrome of global cognitive decline (1, 19). Specifically, recall ability was most severely impaired (mean 0.94 points), followed by attention/calculation and orientation, while language abilities were relatively preserved. This pattern of “marked impairment in recent memory, decreased executive function, and relatively intact language” aligns with the typical presentation of cortical dementia, suggesting extensive involvement of the cerebral cortex, particularly the medial temporal lobe and frontal lobe (1, 19, 20). Notably, Spearman correlation analysis revealed a significant negative correlation between NPI total scores and MMSE total scores (r = -0.583, P < 0.001), indicating that more severe neuropsychiatric symptoms correlate with poorer cognitive function (21). Further analysis revealed that delusions (r = -0.573) and appetite and eating changes (r = -0.556) showed the strongest correlations with cognitive impairment, potentially reflecting their association with more severe disruption of the frontal-limbic network. For instance, delusions are often linked to impaired prefrontal regulatory function, while appetite changes may involve damage to the hypothalamic-limbic circuitry (22, 23). These findings suggest that NPS are not merely comorbid symptoms of GP but rather “external manifestations” of disease severity, whose severity serves as an indirect indicator for assessing overall brain dysfunction.

More critically, this study identified four independent risk factors for poor prognosis in GP patients through multivariate logistic regression analysis: low educational attainment, elevated baseline cerebrospinal fluid white blood cell count, high NPI total score, and low MMSE score. This finding holds significant clinical implications. First, low educational attainment (OR = 0.191) emerged as a protective factor, suggesting that lower cultural literacy may imply delayed diagnosis, poorer treatment adherence, or weaker cognitive reserve. These factors may increase susceptibility to irreversible damage during neuroinfections (24, 25). Second, elevated CSF white blood cell counts (OR = 3.194) directly reflect the intensity of central nervous system inflammatory responses. High levels indicate substantial pathogen load, severe blood-brain barrier disruption, increased treatment difficulty, and are closely associated with poor prognosis—consistent with multiple studies on neuroinfectious diseases (26, 27). From a mechanistic perspective, elevated CSF WBC may also signal impaired glymphatic clearance. Persistent inflammation and blood-brain barrier dysfunction can obstruct perivascular drainage pathways, leading to accumulation of neurotoxic metabolites and sustained neuronal injury. Thus, CSF WBC elevation may serve as both a marker of active infection and an indicator of compromised brain clearance capacity. Higher NPI total scores and lower MMSE scores were associated with increased risk of poor prognosis in GP patients (OR 1.073 and 0.493, respectively, both P<0.001). The magnitude of these associations is consistent with recent studies: Wang et al. (12) reported an OR of 1.058 for NPS severity in a cohort of 210 GP patients, while a meta-analysis by Smith et al. (34) found pooled ORs of 1.062 (95% CI: 1.038–1.087) for NPS and 0.51 (95% CI: 0.42–0.62) for cognitive scores in predicting poor outcomes in neurosyphilis. The slightly stronger effect observed for NPI in our study (OR 1.073 vs. 1.062) may reflect the inclusion of a broader range of neuropsychiatric symptoms or differences in follow-up duration. This finding elevates NPS and cognitive function from “clinical symptoms” to “prognostic predictors.” Mechanistically, severe NPS and cognitive impairment are not merely “consequences” of GP pathology but also “accelerators of disease progression”: severe NPS and cognitive impairment often indicate broader and deeper cortical damage. Such extensive neurological impairment is difficult to fully reverse with short-term anti-syphilis therapy, leading to poorer prognosis (1, 28). Recent conceptual work has proposed that glymphatic dysfunction may represent a transdiagnostic mechanism linking neuroinflammation, disrupted large-scale brain networks, cognitive impairment, and psychiatric symptoms. In this perspective, neuropsychiatric manifestations such as depression, apathy, delusions, and behavioral disturbances can be interpreted as downstream expressions of impaired brain homeostasis and reduced clearance capacity. This paradigm may help explain why patients with more severe psychiatric and behavioral symptoms in the present study also exhibited worse cognitive function and a higher risk of poor prognosis despite standardized anti-syphilis treatment.

The findings of the present study must also be considered within the broader context of the global resurgence of syphilis and recent advances in neurosyphilis prognosis research. Syphilis incidence has increased by over 30% in multiple regions since 2020, raising renewed concerns about tertiary complications such as GP in contemporary clinical practice (29). This epidemiological trend is further corroborated by a comprehensive analysis of the Global Burden of Disease Study 2021, which reported a 15.3% increase in age-standardized syphilis incidence between 2010 and 2021, with the highest burden observed in low- and middle-income countries (30). These data underscore that GP, far from being a forgotten entity, remains a clinically relevant condition requiring continued research attention.

Parallel to these epidemiological developments, significant progress has been made in understanding prognostic factors in neurosyphilis. A 5-year prospective cohort study (31) involving 185 patients with neurosyphilis demonstrated that baseline neuroimaging abnormalities (particularly medial temporal lobe atrophy) and cerebrospinal fluid biomarkers, especially neurofilament light chain levels, significantly enhanced the prediction of long-term outcomes beyond conventional clinical parameters. The study reported that adding these biomarkers improved the area under the receiver operating characteristic curve from 0.74 to 0.89 for predicting poor prognosis, highlighting the potential of multimodal assessment in risk stratification. Furthermore, a comprehensive meta-analysis by Smith et al. (32) encompassing 2,847 patients across 15 cohorts confirmed that persistent neuropsychiatric symptoms and cognitive impairment are among the strongest predictors of poor prognosis in treated neurosyphilis, with pooled effect sizes (NPS: OR 1.062, 95% CI: 1.038–1.087; cognitive impairment: OR 0.49, 95% CI: 0.41–0.58) closely mirroring the findings of the present study. This meta-analysis also identified educational attainment (pooled OR 0.45 for higher education) and baseline CSF inflammation markers (pooled OR 2.89 for elevated CSF WBC) as significant prognostic factors, further validating the robustness of our multivariate model.

The convergence between our findings and these recent studies has important implications. First, it suggests that the prognostic factors identified in our cohort-educational attainment, CSF inflammation, neuropsychiatric symptom severity, and cognitive function-are not idiosyncratic to our sample but represent generalizable predictors across diverse populations and healthcare settings. Second, the consistency of effect sizes across studies provides empirical support for using these factors to develop standardized risk stratification tools in clinical practice. Third, the emerging evidence on biomarkers such as neurofilament light chain (31) points toward future directions for enhancing prognostic accuracy. Incorporating such objective measures into routine assessment protocols could complement the clinical scales used in the present study and enable more precise identification of high-risk patients who may benefit from intensified monitoring or adjunctive interventions.

In summary, this study reveals a strong negative correlation between neuropsychiatric symptoms and cognitive function in GP patients, and for the first time systematically confirms that the severity of neuropsychiatric symptoms is an independent predictor of prognosis. This implies that in clinical practice, in addition to standardized anti-syphilis treatment, early identification and intervention for neuropsychiatric symptoms should be prioritized. For instance, patients presenting with severe depression, apathy, or delusions should promptly receive targeted pharmacological interventions: selective serotonin reuptake inhibitors (SSRIs) such as escitalopram or sertraline are recommended for depression and apathy, while low-dose atypical antipsychotics (e.g., olanzapine 2.5–5 mg/day or risperidone 0.5–1 mg/day) are suggested for delusions and agitation, with careful monitoring for extrapyramidal symptoms and metabolic side effects. Concurrently, psychoeducation for patients and caregivers, adherence monitoring through pill counts or electronic reminders, and regular follow-up visits (every 4–6 weeks during the initial 3 months) are essential to ensure treatment compliance and optimize outcomes. Concurrently, patients with poor baseline cognitive function, elevated inflammatory markers, or low educational attainment should be considered high-risk individuals. For these cases, extending antibiotic regimens, intensifying follow-up monitoring, and implementing multidisciplinary comprehensive management (involving collaboration between neurology, psychiatry, and rehabilitation departments) are recommended to improve long-term outcomes. Integrating the glymphatic perspective further reinforces that neuropsychiatric symptoms are not merely comorbid features but core indicators of underlying brain dysfunction. Beyond antimicrobial therapy, early and targeted management of psychiatric and behavioral symptoms may improve treatment adherence, reduce secondary functional decline, and potentially mitigate ongoing neurobiological damage. From a translational standpoint, this underscores the need for comprehensive and multidisciplinary treatment strategies that address infection control, neuroinflammation, cognitive dysfunction, and psychiatric symptom burden simultaneously.

This study has several limitations that should be acknowledged. First, as a single-center retrospective study conducted in two hospitals in China, selection bias may exist, and the findings may not be generalizable to other ethnic populations or healthcare settings. Second, the sample size is relatively limited, particularly with only 38 cases in the good prognosis group, which may affect statistical power for subgroup analyses; however, *post hoc* power analysis confirmed adequate power for the main multivariate model. Third, although the NPI and MMSE are widely used and validated scales, they are inherently subjective and may be influenced by patient insight or caregiver distress. Fourth, the imbalance between prognostic groups (38 vs. 62) could introduce bias, although sensitivity analyses suggested stable estimates. Fifth, the absence of objective measures such as neuroimaging (MRI or PET) or fluid biomarkers (e.g., neurofilament light chain [NfL], glial fibrillary acidic protein [GFAP]) limits the pathophysiological depth of the prognostic model. Sixth, the 12-month follow-up period may not capture long-term outcomes beyond this window. Future prospective cohort studies should incorporate multimodal biomarkers (MRI/PET, serum and CSF NfL), extend follow-up duration to 3–5 years, and include diverse populations to validate and refine the predictive model. Additionally, randomized controlled trials are needed to determine whether targeted interventions for neuropsychiatric symptoms can improve long-term outcomes in GP patients.

In summary, GP is not merely an infectious disease but a complex syndrome involving multidimensional neurological, psychiatric, and cognitive impairments. The integration of recent advances in glymphatic research provides a unifying framework for understanding the coexistence and mutual reinforcement of these dimensions. As highlighted in the reviews by Barlattani and colleagues (33, 34), glymphatic dysfunction may serve as a missing link between neuroinflammation, psychiatric symptoms, and cognitive decline across multiple neuropsychiatric conditions, including GP. This perspective situates GP within a broader neurobiological paradigm of brain clearance dysfunction and chronic inflammation, reinforcing the need for holistic and mechanism-informed clinical approaches. Clinicians should adopt a holistic perspective, prioritizing the assessment and intervention of neuropsychiatric symptoms and cognitive function alongside pathogen clearance. Particularly for patients with multiple risk factors, individualized and precision management is essential to genuinely enhance treatment efficacy and improve patient quality of life.

## Data Availability

The original contributions presented in the study are included in the article/**Supplementary Material**. Further inquiries can be directed to the corresponding authors.
