# Supplementary material for: Correlation analysis of psychiatric and behavioral symptoms with cognitive impairment and prognosis in patients with general paresis
Source: Front Psychiatry. 2026 Apr 16;17:1769770. doi: 10.3389/fpsyt.2026.1769770 (PMC13128575; doi:10.3389/fpsyt.2026.1769770)
Supplement: Supplementary file 1 [file Table1.pdf]

Supplementary Table S1: Complete Univariate Logistic Regression Analysis of Factors Associated with Poor Prognosis in GPI Patients

Variables	B	SE	Wald χ^2	P-value	Unadjusted OR	95% CI
Age (years)	0.012	0.033	0.132	0.716	1.012	0.948~1.082
Gender (Male vs. Female)	-0.238	0.592	0.162	0.687	0.788	0.247~2.510
Educational Attainment			8.876	0.012		
Elementary school or below	Reference				1.000	
Junior high to high school	-0.712	0.341	4.358	0.037	0.491	0.252~0.957
College or above	-1.156	0.422	7.506	0.006	0.315	0.138~0.719
Marital Status (Married vs. Unmarried/Divorced/Widowed)	-0.114	0.479	0.057	0.812	0.892	0.349~2.283
CSF White Blood Cell Count (×10^6 /L)	0.651	0.186	12.248	<0.001	1.917	1.332~2.761
NPI Total Score	0.065	0.01	25.00	<0.00	1.067	1.040~1.0

		3	0	1		96
MMSE Total Score	-0.669	0.133	25.307	<0.001	0.512	0.394~0.666
